# Energy-Efficient Joint Design of Fronthaul and Edge Links for Cache-Aided C-RAN Systems with Wireless Fronthaul

**DOI:** 10.3390/e21090860

**Published:** 2019-09-03

**Authors:** Junbeom Kim, Daesung Yu, Seung-Eun Hong, Seok-Hwan Park

**Affiliations:** 1 Division of Electronic Engineering, Chonbuk National University, Jeonju 54896, Korea; 2 Electronics and Telecommunications Research Institute (ETRI), Daejeon 34129, Korea

**Keywords:** energy efficiency, C-RAN, edge caching, wireless fronthaul, soft-transfer, hard-transfer, connectivity level

## Abstract

This work addresses the joint design of fronthaul and edge links for a cache-aided cloud radio access network (C-RAN) system with a wireless fronthaul link. Motivated by the fact that existing techniques, such as C-RAN and edge caching, come at the cost of increased energy consumption, an energy efficiency (EE) metric is defined and adopted as the performance metric for optimization. As the fronthaul links can be used to transfer quantized and precoded baseband signals or hard information of uncached files, both soft- and hard-transfer fronthauling strategies are considered. Extensive numerical results validate the impact of edge caching, as well as the advantages of the energy-efficient design over the spectrally-efficient scheme. Additionally, the two fronthauling strategies—the soft- and hard-transfer schemes—are compared in terms of EE.

## 1. Introduction

It has been envisioned that the cloud radio access network (C-RAN) architecture will be able to meet the ever-increasing traffic demands of future wireless communication systems, by migrating baseband signal processing functionalities from base stations, or remote radio heads (RRHs), to a cloud processor (CP) or baseband processing unit (BBU) [1,2].In particular, improved spectral efficiency is expected to be achieved with the C-RAN architecture, thanks to centralized baseband processing at CPs. One of major challenges of implementing C-RAN systems is the overhead on the fronthaul links connecting CPs and RRHs [3,4,5,6,7,8,9,10]. The overhead becomes particularly serious when one adopts cost-effective wireless fronthaul links (see, e.g., [11,12,13,14,15,16,17,18,19]).

The overhead, or capacity requirements, of the fronthaul links in C-RAN systems can be alleviated by adopting narrowband IoT (NB-IoT) technology, which supports low-power and low-cost devices [20], or by adding edge caching functionalities to RRHs [21,22,23,24,25,26,27,28]. The idea of the latter is that the RRHs are equipped with local caches at which popular content frequently requested by mobile user equipment (UE) are pre-fetched, such that the amount of data delivered over the fronthaul links can be reduced at the delivery phase. As in [27], we refer to RRHs equipped with caches as edge nodes (ENs). In [27], an information theoretic analysis of cache-aided C-RAN systems was addressed. In [21,22,23,24,25,26,28] cache-aided C-RAN systems were studied in the aspects of signal processing. Specifically, signal processing optimization for the delivery phase for fixed pre-fetching strategies was discussed in [23,24,25,26,28]. We also mention that, as implementing caching at the edge of the network may face drawbacks, due to limited cache sizes and user mobility from one cell to another, a co-operative hierarchical caching approach has been studied in [29]. In this work, we focus on the design of the delivery phase for a fixed pre-fetching strategy and leave the joint design of co-operative caching and delivery strategies as an interesting future research direction.

All of the techniques discussed above, such as C-RAN and edge caching, come at the cost of increased energy consumption [30,31], which causes increases both in the resulting carbon footprint and system costs. In order to address this issue, it is important to design systems by adopting energy efficiency (EE), instead of spectral efficiency, as the performance metric (as in, e.g., [28,30,31,32,33,34]). The energy-efficient design of C-RAN systems with wired fronthaul links has been addressed in [31,34], and [28] extended such design to the case with edge caching and imperfect channel state information (CSI). The EE metric has also been adopted as the evaluation criterion in [35,36,37] for the design of wireless powered mobile computing systems, reliable routing for wireless ad hoc networks, and MAC strategies for data dissemination. We also note that the C-RAN technology has been recently used for cloud-aided cognitive ambient back-scatter wireless sensor networks, with the aim of providing energy-efficient communications [38].

**Figure 1:**
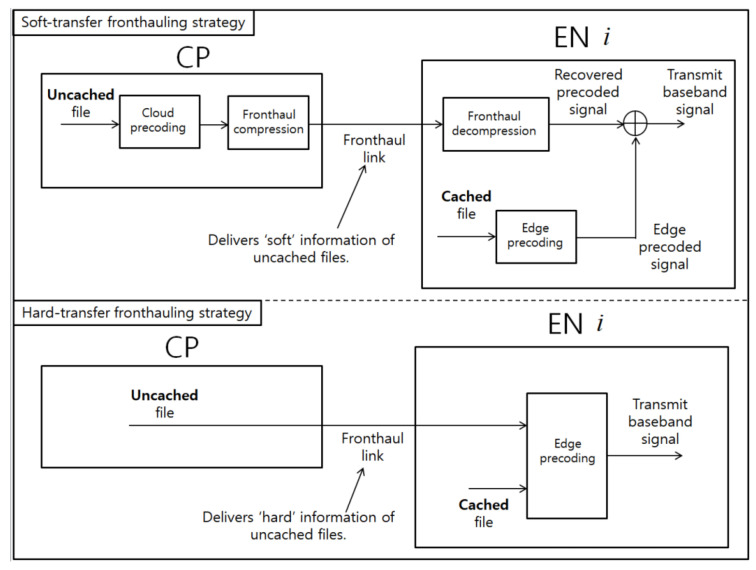
Illustration of soft-transfer and hard-transfer fronthauling strategies.

In this work, we propose an energy-efficient design of a cache-aided C-RAN system equipped with wireless (instead of wired) fronthaul links. We address the design under two different fronthauling strategies: Soft-transfer and hard-transfer schemes (see, e.g., [26,27]). As illustrated in Figure 1, these two fronthauling strategies differ in the type of information delivered over the fronthaul links. Under the soft-transfer (also referred to as compression-based) fronthauling strategy, as in the traditional C-RAN functional split (see, e.g., [8,13,15,26,34]), the CP performs precoding of the files that are requested by UEs but which are uncached by ENs, and quantizes and compresses the precoded baseband signals. Then, the CP sends the bit-streams describing the quantized results to the ENs over the fronthaul link. The information delivered over the fronthaul link is called soft information, as it is a processed version of the uncached files (see, e.g., [26,27]). In contrast, the hard-transfer (or data sharing-based) fronthauling strategy (see, e.g., [8,17,25,26,34]),the fronthaul link delivers the uncached files in raw form, referred to as hard information, to the requesting ENs, where the files are locally processed jointly with the cached files. Signal processing optimization under these two fronthauling strategies has been tackled in [26] for a cache-aided C-RAN system with wired fronthaul links. Furthermore, ref. [27] compared the two fronthauling strategies, in terms of the tradeoff between the cache size at the ENs and the delivery latency.

We tackle the joint design of CP-to-EN fronthaul and EN-to-UE edge links with the goal of maximizing the EE performance while satisfying the CP and per-EN transmission power constraints under the discussed fronthauling strategies. Due to the non-convexity of the formulated problems, we convert the problems into difference-of-convex (DC) problems by change of variables and rank relaxation. To tackle each of the obtained DC problems, we propose a concave-convex procedure (CCCP)-based iterative algorithm which converges to a suboptimal solution. Through analysis of the extensive numerical results, we show that the proposed energy-efficient design provides an EE gain over spectrally-efficient design which increases with the system signal-to-noise ratios (SNRs) and the number of ENs. Furthermore, we show that the hard-transfer fronthauling scheme outperforms the soft-transfer scheme in overall simulated set-ups, as the former is more effective in utilizing the wireless fronthaul resources for multicasting to multiple ENs, which may request overlapping uncached files.

Main contribution of this work is as follows: We tackle, for the first time, the energy-efficient joint design of the fronthaul and edge links for cache-aided C-RAN systems with wireless fronthaul links. Although there are some related works that have studied the impact of edge caching, wireless fronthaul, energy-efficient design and the comparison between the soft- and hard-transfer fronthauling strategies, these factors have not been considered jointly in the literature, as summarized in Table 1. By tackling the challenging optimization problems and through extensive numerical results, we observe the advantages of the energy-efficient design, hard-transfer fronthauling scheme, and the joint design of fronthaul and edge links, in terms of EE performance, in the considered system. Specifically, we show that the hard-transfer fronthauling scheme has the potential for better leveraging the overlapping nature of the uncached files that need to be delivered to the ENs over wireless fronthaul links.

The paper is organized as follows: In Section 2, we describe the system model for a cache-aided C-RAN system with a wireless fronthaul link and define the EE metric. In Section 3, we describe the operations of the CP, ENs, and UEs under the soft-transfer fronthauling strategy; then, the joint optimization of the fronthaul and edge links is tackled. The operations and optimization under the hard-transfer fronthauling scheme are discussed in Section 4. We provide, in Section 5, numerical results validating the advantages of the proposed energy-efficient designs for the soft- and hard-transfer schemes. The paper is concluded in Section 6.

We summarize some notation used throughout the paper, as follows: We denote by I(X;Y) the mutual information between two random variables *X* and *Y*. A circularly symmetric complex Gaussian distribution with mean vector μ and covariance matrix Σ is denoted by CN(μ,Σ). We define $C^{M\times N}$ as the set of all M×N complex matrices, and E(·) stands for expectation. The Hermitian transpose, determinant, and trace operations are denoted by ${\left(\cdot \right)}^{H}$, det(·), and tr(·), respectively, and rank(·) denotes the rank of the input matrix. We denote an M×M identity matrix and an M×N zero matrix, whose elements are filled with 0, as $I_{M}$ and $0_{M\times N}$, respectively. We denote the OR operation by (·|·) for binary input variables.

## 2. System Model

**Figure 2:**
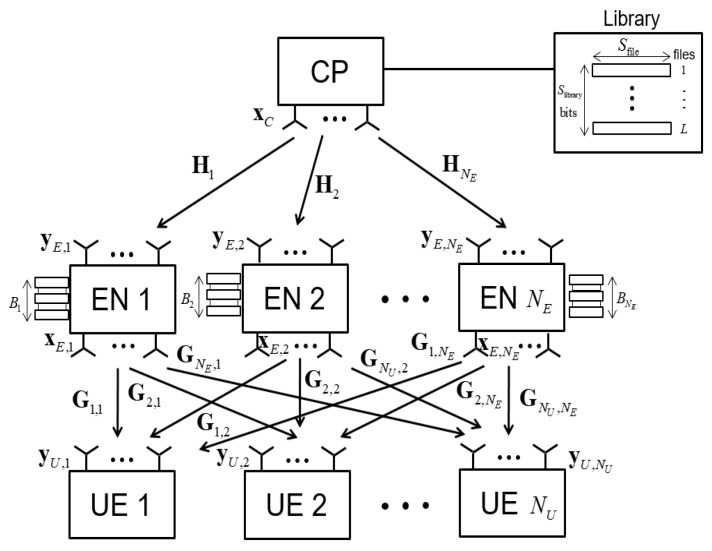
Cache-aided C-RAN system with wireless fronthaul link.

We consider the downlink of a cache-aided C-RAN system where, as shown in Figure 2, there is a CP that communicates with $N_{U}$ UEs through $N_{E}$ ENs. We denote the numbers of antennas of CP, EN *i*, and UE *k* by $n_{C}$, $n_{E,i}$, and $n_{U,k}$, respectively. Here, we assume that each EN *i* uses the same number $n_{E,i}$ of antennas for both reception and transmission on the fronthaul and edge links. However, this discussion can be easily generalized to asymmetric cases. We define $n_{E}=\sum_{i\in N_{E}}n_{E,i}$ and $n_{U}=\sum_{k\in N_{U}}n_{U,k}$, where $N_{E}=\left\{1,2,\ldots ,N_{E}\right\}$ and $N_{U}=\left\{1,2,\ldots ,N_{U}\right\}$ denote the sets of indices of the ENs and UEs, respectively. Each EN *i* is equipped with a local cache of size $B_{i}$ bits, to which popular contents can be pre-fetched to reduce the fronthaul overhead.

### 2.1. Content-Based Communication and Multicast Groups

As in [26,39], we consider a content-based multicast scenario. Accordingly, each UE *k* independently requests a file $f_{l_{k}}$ from a library $F=\{f_{1},f_{2},\ldots f_{L}\}$, where each file is of $S_{\mathrm{file}}$ bits and $l_{k}\in L≜\left\{1,2,\ldots ,L\right\}$ denotes the index of the file requested by UE *k*. Therefore, the library F has $S_{\mathrm{library}}=LS_{\mathrm{file}}$ bits. We assume that the CP can access the library F with a negligible delay. As the UE requests can overlap each other, we define multicast groups. We first define the set of indices of the requested files as $L_{\mathrm{req}}=\cup_{k\in N_{U}}\left\{l_{k}\right\}$, and $L_{\mathrm{req}}=\left|L_{\mathrm{req}}\right|$ distinct indices in $L_{\mathrm{req}}$ as ${\tilde{l}}_{1},{\tilde{l}}_{2},\ldots {\tilde{l}}_{L_{\mathrm{req}}}$. Defining the *g*th multicast group as the set of UEs that request file $f_{{\tilde{l}}_{g}}$ (i.e., $N_{U,g}=\left\{k\in N_{U}|l_{k}={\tilde{l}}_{g}\right\}$), the system has $L_{\mathrm{req}}$ multicast groups. We denote by $G_{U}=\left\{1,2,\ldots ,L_{\mathrm{req}}\right\}$ the set of the indices of the multicast groups. We assume that the $L_{\mathrm{req}}$ multicast groups have equal priority and, hence, they are communicated from the CP to the requesting UEs at the same data rate of *R* bits per symbol.

### 2.2. Edge Caching

During the off-peak traffic period, each EN *i* can pre-fetch some popular contents which are frequently requested by UEs to its local cache (of size $B_{i}$ bits). As the ENs typically have lighter structures than the CP, it is reasonable to assume that $B_{i}\leq S_{\mathrm{library}}$. We model the pre-fetching strategy by defining a binary caching variable

(1) $$c_{i,l}=\left\{\begin{array}{cc} 1, & \mathrm{if}\;\;\mathrm{file}\;\;f_{l}\;\;\mathrm{is}\;\;\mathrm{cached}\;\;\mathrm{by}\;\;\mathrm{EN}\;\;i\\ 0, & \mathrm{otherwise} \end{array}\right..$$

We note that the caching variables $c={\left\{c_{i,l}\right\}}_{i\in N_{E},l\in L}$ cannot be determined in adaptation to instantaneous file requests or CSI, as the pre-fetching takes place during off-peak traffic periods. In this work, we assume that the caching variables c are arbitrarily pre-fixed, and the joint design of co-operative pre-fetching and delivery strategies is left as a future work.

### 2.3. Wireless Channel Models of Fronthaul and Edge Links

We assume that the CP-to-EN fronthaul link is orthogonal to the EN-to-UE edge link. Therefore, the two links do not interfere with each other. Also, we consider flat fading channel models for both the CP-to-EN fronthaul and EN-to-UE edge links. Then, the baseband received signal $y_{E,i}\in C^{n_{E,i}\times 1}$ of EN *i* on the wireless fronthaul link is given as

(2) $$y_{E,i}=H_{i}x_{C}+z_{E,i},$$

where $x_{C}\in C^{n_{C}\times 1}$ denotes the transmitted signal of the CP, which is subject to the power constraint $E||x_{C}{\left|\right|}^{2}\leq P_{C}$, where $H_{i}\in C^{n_{E,i}\times n_{C}}$ represents the channel matrix from the CP to EN *i* and $z_{E,i}∼\mathrm{CN}\left(0,\sigma_{E}^{2}I_{n_{E,i}}\right)$ denotes the additive white Gaussian noise (AWGN) vector at EN *i*.

Each EN *i* processes the received signal $y_{E,i}$, generating a baseband signal $x_{E,i}\in C^{n_{E,i}\times 1}$ which is transmitted over EN-to-UE edge links. We impose a transmission power constraint on $x_{E,i}$ as $E||x_{E,i}{\left|\right|}^{2}\leq P_{E,i}$. The received signal $y_{U,k}\in C^{n_{U,k}\times 1}$ of UE *k* on the edge link can be written as

(3) $$y_{U,k}=\sum_{i\in N_{E}}G_{k,i}x_{E,i}+z_{U,k}=G_{k}x_{E}+z_{U,k},$$

where $G_{k,i}\in C^{n_{U,k}\times n_{E,i}}$ represents the channel matrix from EN *i* to UE *k*, $z_{U,k}∼\mathrm{CN}\left(0,\sigma_{U}^{2}I_{n_{U,k}}\right)$ denotes the AWGN vector at UE *k*, $G_{k}=\left[G_{k,1}G_{k,2}\cdots G_{k,N_{E}}\right]$ represents the channel matrix from all ENs to UE *k*, and $x_{E}={\left[x_{E,1}^{H}x_{E,2}^{H}\cdots x_{E,N_{E}}^{H}\right]}^{H}$ is transmitted signal of all ENs.

In this work, we assume that the CP has perfect CSI ${\left\{H_{i}\right\}}_{i\in N_{E}}$ and ${\left\{G_{k,i}\right\}}_{k\in N_{U},i\in N_{E}}$ and manages the operation of all nodes (i.e., the CP, ENs, and UEs). The analysis of the impact of imperfect CSI and robust design, taking into account the CSI error, are left as future work.

### 2.4. Energy Efficiency Metric

As illustrated in Section 1, our goal is to address the energy-efficient design for the described cache-aided C-RAN system with wireless fronthaul link. The EE metric, denoted by Θ, is defined as the data rate *R* that can be supported per unit power [31,32,33,34]. Mathematically, we write Θ as

(4) $$\Theta =\frac{R}{E{∥x_{C}∥}^{2}+\eta_{C}+\sum_{i\in N_{E}}\left(E{∥x_{E,i}∥}^{2}+\eta_{E,i}\right)},$$

where $\eta_{C}$ and $\eta_{E,i}$ represent the constant circuit powers consumed at the CP and EN *i*, respectively; regardless of the radio-frequency (RF) transmission. As the constraints on the achievable rate *R* are formulated differently, depending on the fronthaul transmission mode, we will clarify the constraints under the soft-transfer and hard-transfer fronthauling modes in Section 3 and Section 4, respectively.

## 3. Energy-Efficient Design Under Soft-Transfer Fronthaul Mode

In this section, we discuss the energy-efficient design of the cache-aided C-RAN system described in Section 2 under the soft-transfer fronthauling scheme, whereby the fronthaul links carry quantized and precoded versions of the uncached files. The detailed operation is described in Section 3.1, Section 3.2, Section 3.3 and Section 3.4, and the optimization will be discussed in Section 3.5.

### 3.1. Fronthaul SDMA Precoding

The CP needs to first establish communication links to the ENs to send soft information of uncached files (which will be detailed in the next subsection). For communication to ENs, time division multiple access (TDMA) was considered in [14,16], while the works [12,13,15,17] assumed space time division multiple access (SDMA) fronthaul beamforming (or precoding) techniques. In this work, we assume the latter, as it is more efficient when the CP uses sufficiently many antennas.

To elaborate on fronthaul SDMA precoding, we denote by $s_{E,i}\in C^{d_{E,i}\times 1}$ the data signal which encodes the soft information to be delivered to EN *i*. The number $d_{E,i}$ of data streams should satisfy the condition $d_{E,i}\leq \mathrm{rank}\left(n_{C},n_{E,i}\right)$, and we assume a Gaussian channel codebook; that is, $s_{E,i}∼\mathrm{CN}\left(0,I_{d_{E,i}}\right)$. With SDMA fronthaul precoding, the transmitted signal of the CP is given as

(5) $$x_{C}=\sum_{i\in N_{E}}F_{i}s_{E,i},$$

where $F_{i}\in C^{n_{C}\times d_{E,i}}$ represents the precoding matrix for $s_{E,i}$. With (5), the CP power constraint can be rewritten as

(6) $$\sum_{i\in N_{E}}\mathrm{tr}\left(F_{i}F_{i}^{H}\right)\leq P_{C}.$$

If we assume that EN *i* decodes the signal $s_{E,i}$ based on the received signal $y_{E,i}$ without interference decoding, the fronthaul rate, $C_{i}$, at which the CP can communicate with EN *i* in bits per symbol, is constrained as

(7) $$\begin{array}{cc} C_{i}\leq & f_{E,i}\left(F\right)≜I\left(s_{E,i};y_{E,i}\right)\\ = & \log_{2}\det\left(\sum_{j\in N_{E}}H_{i}F_{j}F_{j}^{H}H_{i}^{H}+\sigma_{E}^{2}I_{n_{E,i}}\right)-\log_{2}\det\left(\sum_{j\in N_{E}\setminus \left\{i\right\}}H_{i}F_{j}F_{j}^{H}H_{i}^{H}+\sigma_{E}^{2}I_{n_{E,i}}\right), \end{array}$$

with the notation $F≜{\left\{F_{i}\right\}}_{i\in N_{E}}$.

### 3.2. Cloud Precoding and Fronthaul Compression

In order to improve the performance of the multicast transmission on the EN-to-UE edge links, the CP performs cloud precoding of uncached files for each EN *i* by

(8) $${\tilde{x}}_{E,i}=\sum_{g\in G_{U}}{\bar{c}}_{i,{\tilde{l}}_{g}}U_{i,g}s_{U,g},$$

where $U_{i,g}\in C^{n_{E,i}\times d_{U,g}}$ is the precoding matrix applied to the signal $s_{U,g}\in C^{d_{U,g}\times 1}∼\mathrm{CN}\left(0,I_{d_{U,g}}\right)$ which encodes the file $f_{{\tilde{l}}_{g}}$ not available at EN *i* with $c_{i,{\tilde{l}}_{g}}=0$. For a binary variable *c*, we define $\bar{c}=1-c$. The number $d_{U,g}$ of data streams for the *g*th multicast group is set to satisfy the condition $d_{U,g}=\min\left\{n_{E},\;\min_{k\in N_{U,g}}n_{U,k}\right\}$.

To send the precoded signal ${\tilde{x}}_{E,i}$ to EN *i* over the fronthaul link of capacity $C_{i}$ given in (7), the CP performs fronthaul quantization and compression on ${\tilde{x}}_{E,i}$. As in the related works [4,5,6,7,8,9,10] on fronthaul compression, we model the output signal of the compression as

(9) $${\hat{x}}_{E,i}={\tilde{x}}_{E,i}+q_{E,i},$$

where $q_{E,i}$ represents the distortion signal caused by the quantization. Following the rate-distortion theoretic approaches considered in the related works [4,5,6,7,8,9,10,13,15], we assume a Gaussian quantization codebook under which the quantization noise signal $q_{E,i}$ is independent of the source signal ${\tilde{x}}_{E,i}$ and distributed as $q_{E,i}∼\mathrm{CN}\left(0,\Omega_{i}\right)$. The output signal ${\hat{x}}_{E,i}$ can be reliably delivered to EN *i* if the following condition is met:

(10) $$\begin{array}{cc} g_{i}\left(U,\Omega \right) & ≜I\left({\tilde{x}}_{E,i};{\hat{x}}_{E,i}\right)\\ \; & =\log_{2}\det\left(\sum_{g\in G_{U}}{\bar{c}}_{i,{\tilde{l}}_{g}}U_{i,g}U_{i,g}^{H}+\Omega_{i}\right)-\log_{2}\det\left(\Omega_{i}\right)\leq C_{i}, \end{array}$$

with the notations $U≜{\left\{U_{i,g}\right\}}_{i\in N_{E},g\in G_{U}}$ and $\Omega ≜{\left\{\Omega_{i}\right\}}_{i\in N_{E}}$.

### 3.3. Edge Precoding and Superposition Coding

Among the requested files ${\left\{f_{l}\right\}}_{l\in L_{\mathrm{req}}}$, each EN *i* can locally process the files ${\left\{f_{l}\right\}}_{l\in L_{\mathrm{req}},c_{i,l}=1}$ that are pre-fetched at its local cache. Therefore, we assume that EN *i* sends a superposition of the quantized signal ${\hat{x}}_{E,i}$, which was received on the wireless fronthaul link, and a locally precoded signal, written as $\sum_{g\in G_{U}}c_{i,{\tilde{l}}_{g}}V_{i,g}s_{U,g}$. Here, $V_{i,g}\in C^{n_{E,i}\times d_{U,g}}$ and $s_{U,g}∼\mathrm{CN}\left(0,I_{d_{U,g}}\right)$ represent the precoder matrix and the data signal, respectively, for the file $f_{{\tilde{l}}_{g}}$. As a result, the transmitted signal $x_{E,i}$ of EN *i* is given as

(11) $$\begin{array}{cc} x_{E,i} & ={\hat{x}}_{E,i}+\sum_{g\in G_{U}}c_{i,{\tilde{l}}_{g}}V_{i,g}s_{U,g}\\ \; & =\sum_{g\in G_{U}}T_{i,g}s_{U,g}+q_{E,i}, \end{array}$$

where we have defined the effective precoding matrix $T_{i,g}={\bar{c}}_{i,{\tilde{l}}_{g}}U_{i,g}+c_{i,{\tilde{l}}_{g}}V_{i,g}$ for the *g*th multicast group at EN *i*. With the precoding model (11), the transmission power constraint at EN *i* can be rewritten as

(12) $$\sum_{g\in G_{U}}\mathrm{tr}\left(T_{i,g}T_{i,g}^{H}\right)+\mathrm{tr}\left(\Omega_{i}\right)\leq P_{E,i}.$$

### 3.4. Decoding and Achievable Rate

We assume that a UE *k* in the *g*th multicast group (i.e., $k\in N_{U,g}$) tries to decode its requested content $f_{{\tilde{l}}_{g}}$ from the received signal $y_{U,k}$ without decoding the interference signals from the other multicast groups. In order for the explained decoding to be successful, the rate *R* should be bounded by

(13) $$\begin{array}{cc} R\leq & f_{U,g,k}\left(T,\Omega \right)≜I\left(s_{U,g};y_{U,k}\right)\\ = & \log_{2}\det\left(\sum_{g^{\prime }\in G_{U}}G_{k}T_{g^{\prime }}T_{g^{\prime }}^{H}G_{k}^{H}+G_{k}\bar{\Omega }G_{k}^{H}+\sigma_{U}^{2}I_{n_{U,k}}\right)\\ - & \log_{2}\det\;\left(\;\sum_{g^{\prime }\in G_{U}\;\setminus \left\{g\right\}}\;\;\;\;G_{k}T_{g^{\prime }}T_{g^{\prime }}^{H}G_{k}^{H}\;+\;G_{k}\bar{\Omega }G_{k}^{H}+\sigma_{U}^{2}I_{n_{U,k}}\;\right)\;, \end{array}$$

for all $g\in G_{U}$ and $k\in N_{U,g}$, with $T_{g}={\left[T_{1,g}^{H}T_{2,g}^{H}\cdots T_{N_{E},g}^{H}\right]}^{H}$, $T≜{\left\{T_{g}\right\}}_{g\in G_{U}}$, and $\bar{\Omega }=\mathrm{diag}\left(\Omega_{1},\Omega_{2},\ldots ,\Omega_{N_{E}}\right)$.

### 3.5. Optimization

In this subsection, we discuss the joint optimization of the fronthaul SDMA precoding F, the effective cloud and edge precoding T, and the quantization noise covariance matrices Ω with the criterion of maximizing the EE performance Θ, as defined in (4). We can mathematically formulate the mentioned optimization problem as

(14a) $$\begin{array}{cc} \underset{F,T,\Omega ,R}{\mathrm{maximize}}\;\; & \frac{R}{p_{\mathrm{total}}\left(F,T,\Omega \right)} \end{array}$$

(14b) $$\begin{array}{cc} s.t.\;\; & R\;\leq \;f_{U,g,k}\left(T,\Omega \right),\;g\in G_{U},\;k\in N_{U,g}, \end{array}$$

(14c) $$\begin{array}{cc} \; & g_{i}\left(T,\Omega \right)\leq f_{E,i}\left(F\right),\;\;i\in N_{E}, \end{array}$$

(14d) $$\begin{array}{cc} \; & p_{C}\left(F\right)\leq P_{C}, \end{array}$$

(14e) $$\begin{array}{cc} \; & p_{E,i}\left(T,\Omega \right)\leq P_{E,i},\;\;i\in N_{E}. \end{array}$$

In (14), the function $p_{\mathrm{total}}\left(F,T,\Omega \right)$ measures the total power consumption at the CP and the ENs, and is defined as

(15) $$\begin{array}{c} p_{\mathrm{total}}\left(F,T,\Omega \right)=p_{C}\left(F\right)+\eta_{C}+\sum_{i\in N_{E}}\left(p_{E,i}\left(T,\Omega \right)+\eta_{E,i}\right), \end{array}$$

where $p_{C}\left(F\right)≜\sum_{i\in N_{E}}\mathrm{tr}\left(F_{i}F_{i}^{H}\right)$ and $p_{E,i}\left(T,\Omega \right)≜\sum_{g\in G_{U}}\mathrm{tr}\left(T_{i,g}T_{i,g}^{H}\right)+\mathrm{tr}\left(\Omega_{i}\right)$ represent the powers consumed for RF transmissions at the CP and EN *i*, respectively. The function $g_{i}\left(T,\Omega \right)$ in (14c) is obtained by substituting $U_{i,g}\leftarrow T_{i,g}$ for all $g\in G_{U}$ into the function $g_{i}\left(U,\Omega \right)$ defined in (10). We note that the condition (14b), which comes from (13), guarantees successful decoding of the requested files at the UEs, and the constraint (14c), obtained by combining the conditions (7) and (10), imposes reliable decompression of the quantized signals at ENs. The constraints (14d) and (14e) correspond to the transmission power constraints at the CP and ENs, respectively.

It is challenging to solve the problem (14), which is non-convex due to the objective function in (14a) and the constraints (14b) and (14c). We first handle the non-convexity of the objective fractional function by replacing it with a new variable Θ, which is constrained by

(16) $$\Theta \leq \frac{R}{p_{\mathrm{total}}\left(F,T,\Omega \right)}.$$

As the above constraint is still non-convex, we consider the following equivalent constraint obtained by taking the monotonically increasing log function on both sides:

(17) $$\ln\Theta \leq \ln R-\ln\;p_{\mathrm{total}}\left(F,T,\Omega \right).$$

Another way of handling the non-convexity of (16) is to adopt the fractional programming (FP) approach, as in [41]. We leave the comparison between the proposed decoupling and the FP approaches as a future work.

With the described manipulation, we obtain the following problem equivalent to (Section 3.5):

(18a) $$\begin{array}{cc} \underset{F,T,\Omega ,R,\Theta }{\mathrm{maximize}}\;\; & \;\;\Theta \end{array}$$

(18b) $$\begin{array}{cc} s.t.\;\; & \ln\Theta \leq \ln R-\ln\;p_{\mathrm{total}}\left(F,T,\Omega \right), \end{array}$$

(18c) $$\begin{array}{cc} \; & R\;\leq \;f_{U,g,k}\left(T,\Omega \right),\;g\in G_{U},\;k\in N_{U,g}, \end{array}$$

(18d) $$\begin{array}{cc} \; & g_{i}\left(T,\Omega \right)\leq f_{E,i}\left(F\right),\;\;i\in N_{E}, \end{array}$$

(18e) $$\begin{array}{cc} \; & p_{C}\left(F\right)\leq P_{C}, \end{array}$$

(18f) $$\begin{array}{cc} \; & p_{E,i}\left(T,\Omega \right)\leq P_{E,i},\;\;i\in N_{E}. \end{array}$$

Although (18) is still non-convex, we can obtain a DC problem by change of variables ${\tilde{F}}_{i}=F_{i}F_{i}^{H}$ and ${\tilde{T}}_{g}=T_{g}T_{g}^{H}$ and by relaxing the constraints $\mathrm{rank}\left({\tilde{F}}_{i}\right)\leq d_{E,i}$ and $\mathrm{rank}\left({\tilde{T}}_{g}\right)\leq d_{U,g}$. It was reported in, for example, [4,13,15,25,26], that an efficient solution to a DC problem can be found by using the CCCP approach. The main idea of CCCP is to iteratively solve the convex problems that are obtained by linearly approximating the terms that induce the non-convexity of the problem. The approximated convex problem changes over the iterations, as the reference point used for the linear approximation at each step is set to the solution of the convex problem in the previous iteration. The detailed algorithm was derived, in a similar way to those in [4,13,15,25,26], as Algorithm 1. After the CCCP based iterative algorithm converges, the obtained quadratic matrices ${\tilde{F}}_{i}$ and ${\tilde{T}}_{g}$ may not satisfy the rank constraints. We propose to obtain each CP precoding matrix $F_{i}$ with the standard projection approach: $F_{i}\leftarrow Q_{d_{E,i}}\left({\tilde{F}}_{i}\right)\;S_{d_{E,i}}{\left({\tilde{F}}_{i}\right)}^{1/2}$, where $Q_{d}\left(\cdot \right)$ takes the leading *d* eigenvectors of the input matrix as the column vectors and $S_{d}\left(\cdot \right)$ is a diagonal matrix, whose diagonal elements are the *d* leading eigenvalues of the input matrix. Similarly, each effective cloud and edge precoding matrix $T_{g}$ is obtained as $T_{g}\leftarrow Q_{d_{U,g}}\left({\tilde{T}}_{g}\right)\;S_{d_{U,g}}{\left({\tilde{T}}_{g}\right)}^{1/2}$.
**Algorithm 1** CCCP-based algorithm for problem (18)**1.** Initialize the variables $\Theta^{\prime },{\tilde{F}}^{\prime },{\tilde{T}}^{\prime }$ and $\Omega^{\prime }$ that satisfy the constraints of the problem (18). **2.** Update $\Theta^{\prime \prime },{\tilde{F}}^{\prime \prime },{\tilde{T}}^{\prime \prime }$ and $\Omega^{\prime \prime }$ as an optimal solution of the (convex) problem (A1) in Appendix A. **3.** Stop if a convergence criterion is satisfied. Otherwise, set $\Theta^{\prime }\leftarrow \Theta^{\prime \prime },{\tilde{F}}^{\prime }\leftarrow {\tilde{F}}^{\prime \prime },{\tilde{T}}^{\prime }\leftarrow {\tilde{T}}^{\prime \prime }$ and $\Omega^{\prime }\leftarrow \Omega^{\prime \prime }$ and go back to Step 2.

The proposed CCCP algorithm is an instance of the successive convex approximation (SCA) approach [42], whose worst-case order of complexity is given as $O(N_{\mathrm{itr}}\sqrt{N_{\mathrm{const}}}\log\left(N_{\mathrm{const}}/\epsilon \right))$ [43]. Here, $N_{\mathrm{itr}}$ denotes the maximum number of iterations, $N_{\mathrm{const}}$ is the number of constraints of the convex problem (A1) in Appendix A, and ϵ indicates the desired error tolerance. Using simulation, we checked that the algorithm converges within a few tens of iterations for all simulated cases. We will show, in Section 5, the convergence behavior of the algorithm. Furthermore, the number $N_{\mathrm{const}}$ of constraints of (A1) is equal to $N_{\mathrm{const}}=N_{U}+2N_{E}+2$.

## 4. Energy-Efficient Design Under Hard-Transfer Fronthaul Mode

In the soft-transfer fronthauling scheme in Section 3, we used fronthaul SDMA precoding to create orthogonal fronthaul links across ENs, over which the quantized signals ${\left\{{\hat{x}}_{E,i}\right\}}_{i\in N_{E}}$ are communicated. However, this approach may not be efficient, in the sense that the overlapping nature of the files requested by different ENs is not sufficiently leveraged. Motivated by this observation, in this section, we discuss the energy-efficient design under hard-transfer fronthauling mode, whereby the fronthaul links carry hard information of the uncached files.

### 4.1. Connectivity Level and Fronthaul Multicasting

We assume that, under the hard-transfer fronthauling mode, each UE *k* is served by the union of the closest α ENs, which are denoted as $N_{E,k}$, and that the ENs that cache the content $f_{l_{k}}$ requested by UE *k* (i.e., $c_{i,l_{k}}=1$). This means that each UE is served by at least α ENs, where we refer to α as the connectivity level. We note that increasing α has conflicting impacts on the system performance (see also, e.g., [40]): With larger α, the overhead of the wireless fronthaul link will increase, as the ENs need to receive more files from the CP. On the other hand, the inter-group interference signals which occur on the edge link will be better managed when the ENs co-operate with a larger connectivity level α. This suggests that the connectivity level α should be carefully chosen in adaptation to the system environment, such as the channel states ${\left\{H_{i}\right\}}_{i\in N_{E}}$ and ${\left\{G_{k,i}\right\}}_{k\in N_{U},i\in N_{E}}$. In this section, we discuss optimization for fixed α; however, in Section 5, we will show the performance when the optimal value of α is chosen.

Under the hard-transfer fronthauling mode, each EN *i* needs to receive the files that are requested by the nearby UEs *k* with $i\in N_{E,k}$ and not cached by EN *i* from the CP on the fronthaul link. We denote the set of the indices of those files as

(19) $$L_{E,i}=\left\{l_{k},\;k\in N_{U}|i\in N_{E,k}\;\mathrm{and}\;c_{i,l_{k}}=0\right\}.$$

Furthermore, we define the binary transfer variables $u_{i,l}$ as

(20) $$u_{i,l}=\left\{\begin{array}{cc} 1, & \mathrm{if}\;\;l\in L_{E,i}\\ 0, & \mathrm{otherwise} \end{array}\right..$$

Equation (20) means that the variable $u_{i,l}$ takes a value of 1 if the file $f_{i,l}$ needs to be delivered to EN *i* over the fronthaul link and 0 otherwise (i.e., if the file $f_{i,l}$ is pre-fetched at EN *i* or $i\notin N_{E,k}$).

As there could be overlaps among the sets $L_{E,1}$, $L_{E,2}$, …, $L_{E,N_{E}}$, we consider a multicast fronthaul transmission from the CP to the ENs. We define the set $L_{E}=\cup_{i\in N_{E}}L_{E,i}$ of the indices of all the files which are multicast over the fronthaul link. We denote the $L_{E}=\left|L_{E}\right|$ distinct indices in $L_{E}$ as ${\hat{l}}_{1},{\hat{l}}_{2},\ldots ,{\hat{l}}_{L_{E}}$ (i.e., $L_{E}=\left\{{\hat{l}}_{1},{\hat{l}}_{2},\ldots ,{\hat{l}}_{L_{E}}\right\}$). Therefore, we have $L_{E}$ multicast groups for the fronthaul multicast transmission. We denote the set of the ENs corresponding to the *g*th fronthaul multicast group which need to receive the file $f_{{\hat{l}}_{g}}$ over the fronthaul link as $N_{E,g}=\left\{i\in N_{E}|{\hat{l}}_{g}\in L_{E,i}\right\}$. Furthermore, we define the set of the indices of the fronthaul multicast groups as $G_{E}=\left\{1,2,\ldots ,L_{E}\right\}$.

### 4.2. Fronthaul Multicast Precoding

In this subsection, we describe the fronthaul multicast transmission from the CP to the ENs. The CP performs channel encoding on the file $f_{{\hat{l}}_{g}}$ for each fronthaul multicast group $g\in G_{E}$ and obtains a baseband signal $s_{E,g}\in C^{d_{E,g}\times 1}$, distributed as $s_{E,g}∼\mathrm{CN}\left(0,I_{d_{E,g}}\right)$. We set the number $d_{E,g}$ of data streams, such that $d_{E,g}\leq \min\left\{n_{C},\min_{i\in N_{E,g}}n_{E,i}\right\}$ is satisfied.

The CP precodes the encoded signals ${\left\{s_{E,g}\right\}}_{g\in G_{E}}$ so that its transmitted signal $x_{C}$ is given as

(21) $$x_{C}=\sum_{g\in G_{E}}A_{g}s_{E,g},$$

where $A_{g}\in C^{n_{C}\times d_{E,g}}$ is the precoding matrix for the *g*th fronthaul multicast group. With (21), the CP power constraint can be rewritten as

(22) $$\sum_{g\in G_{E}}\mathrm{tr}\left(A_{g}A_{g}^{H}\right)\leq P_{C}.$$

We assume that each EN *i* performs a symbol-by-symbol decoding to recover the files ${\left\{f_{l}\right\}}_{l\in L_{E,i}}$ from the received signal $y_{E,i}$ without decoding the interference signals. Furthermore, since the received signal $y_{E,i}$ may contain the signals that encode the cached files of EN *i*, the EN can exploit its cached contents for known interference cancellation. Under this assumption, the achievable rate for the *g*th fronthaul multicast group, denoted by $R_{E,g}$, is constrained as

(23) $$\begin{array}{cc} R_{E,g} & \leq f_{E,g,i}\left(A\right)\\ \; & ≜I\left(s_{E,g};y_{E,i}|\left\{s_{E,m},\;m\in G_{E}|c_{i,{\hat{l}}_{m}}=1\right\}\right)\\ \; & =\log_{2}\det\left(\sum_{m\in G_{E}}{\bar{c}}_{i,{\hat{l}}_{m}}H_{i}A_{m}A_{m}^{H}H_{i}^{H}+\sigma_{E}^{2}I_{n_{E,i}}\right)\\ \; & -\log_{2}\det\left(\sum_{m\in G_{E}\setminus \left\{g\right\}}{\bar{c}}_{i,{\hat{l}}_{m}}H_{i}A_{m}A_{m}^{H}H_{i}^{H}+\sigma_{E}^{2}I_{n_{E,i}}\right), \end{array}$$

for all $i\in N_{E,g}$, where we have used the notation $A≜{\left\{A_{g}\right\}}_{g\in G_{E}}$.

### 4.3. Edge Multicast Precoding

Once the fronthaul multicast transmission is finished, each EN *i* can process the files $f_{l}$ which have been pre-fetched to its cache (i.e., $c_{i,l}=1$) or have been received from the CP (i.e., $u_{i,l}=1$) for the edge multicast transmission. We assume that EN *i* obtains its transmitted signal $x_{E,i}$ on the edge link by performing an edge multicast precoding as

(24) $$x_{E,i}=\sum_{g\in G_{U}}\left(c_{i,{\tilde{l}}_{g}}|u_{i,{\tilde{l}}_{g}}\right)D_{i,g}s_{U,g},$$

where $D_{i,g}\in C^{n_{E,i}\times d_{U,g}}$ denotes the precoding matrix for the *g*th edge multicast group at EN *i* and $s_{U,g}\in C^{d_{U,g}\times 1}$ represents the baseband signal encoding $f_{{\tilde{l}}_{g}}$ with $d_{U,g}\leq \min\left\{n_{E},\min_{k\in N_{U,g}}n_{U,k}\right\}$ of data streams, and is distributed as $s_{U,g}∼\mathrm{CN}\left(0,I_{d_{U,g}}\right)$. The transmission power constraint for EN *i* under (24) can be stated as

(25) $$\sum_{g\in G_{U}}\left(c_{i,{\tilde{l}}_{g}}|u_{i,{\tilde{l}}_{g}}\right)\mathrm{tr}\left(D_{i,g}D_{i,g}^{H}\right)\leq P_{E,i}.$$

To simplify the notation, we define the effective edge multicast precoding matrix $W_{i,g}=\left(c_{i,{\tilde{l}}_{g}}|u_{i,{\tilde{l}}_{g}}\right)D_{i,g}$ which is constrained by

(26) $$\begin{array}{cc} \; & \mathrm{tr}\left(W_{i,g}W_{i,g}^{H}\right)=0,\;\;\mathrm{if}\;\;c_{i,{\tilde{l}}_{g}}=u_{i,{\tilde{l}}_{g}}=0. \end{array}$$

Using (26), we can write the total transmitted signal $x_{E}={\left[x_{E,1}^{H}\;\cdots \;x_{E,N_{E}}^{H}\right]}^{H}$ of all the ENs on the wireless edge link as

(27) $$x_{E}=\sum_{g\in G_{U}}W_{g}s_{U,g},$$

with the effective edge precoding matrix $W_{g}={\left[W_{1,g}^{H}\;\cdots \;W_{N_{E,}g}^{H}\right]}^{H}$ for the *g*th edge multicast group. We note that the *i*th submatrix $W_{i,g}$ of $W_{g}$ can be expressed as $W_{i,g}=E_{i}^{H}W_{g}$, where the shaping matrix $E_{i}\in C^{n_{E}\times n_{E,i}}$ is defined as

(28) $$E_{i}={\left[0_{\left(\sum_{j=1}^{i-1}n_{E,j}\right)\times n_{E,i}}^{H}\;I_{n_{E,i}}\;\;0_{\left(\sum_{j=i+1}^{N_{E}}n_{E,j}\right)\times n_{E,i}}^{H}\right]}^{H}.$$

We assume that each UE *k* in the *g*th edge multicast group performs single-user decoding to obtain the requested file $f_{{\tilde{l}}_{g}}$, based on the received signal $y_{U,k}$. The achievable rate *R* is, hence, limited by

(29) $$\begin{array}{cc} R & \leq f_{U,g,k}\left(W\right)≜I\left(s_{U,g};y_{U,k}\right)\\ \; & =\log_{2}\det\left(\sum_{g^{\prime }\in G_{U}}G_{k}W_{g^{\prime }}W_{g^{\prime }}^{H}G_{k}^{H}+\sigma_{U}^{2}I_{n_{U,k}}\right)\\ \; & -\log_{2}\det\left(\sum_{g^{\prime }\in G_{U}\setminus \left\{g\right\}}G_{k}W_{g^{\prime }}W_{g^{\prime }}^{H}G_{k}^{H}+\sigma_{U}^{2}I_{n_{U,k}}\right), \end{array}$$

for all $g\in G_{U}$ and $k\in N_{U,g}$, where we have used the notation $W≜{\left\{W_{g}\right\}}_{g\in G_{U}}$.

We note that each *g*th edge multicast file $f_{{\tilde{l}}_{g}}$ needs to also be reliably communicated over the fronthaul link if it belongs to the set of the fronthaul multicast files; that is, ${\tilde{l}}_{g}\in L_{E}$. Therefore, the rate *R* of the edge multicast files has the following additional constraint:

(30) $$R\leq R_{E,g^{\prime }},\;\mathrm{if}\;\;{\tilde{l}}_{g}={\hat{l}}_{g^{\prime }},\;\;g\in G_{U},\;g^{\prime }\in G_{E}.$$

### 4.4. Optimization

We now address the joint optimization of the fronthaul multicast precoding A and the effective edge multicast precoding matrices W, with the aim of maximizing the EE performance under the constraints on the transmision powers at the CP and the ENs. We can state the problem as

(31a) $$\begin{array}{cc} \underset{A,W,R,R_{E}}{\mathrm{maximize}}\;\; & \frac{R}{p_{\mathrm{total}}\left(A,W\right)} \end{array}$$

(31b) $$\begin{array}{cc} s.t.\;\; & R\leq f_{U,g,k}\left(W\right),\;g\in G_{U},\;k\in N_{U,g}, \end{array}$$

(31c) $$\begin{array}{cc} \; & R_{E,g}\leq f_{E,g,i}\left(A\right),\;\;g\in G_{E},\;i\in N_{E,g}, \end{array}$$

(31d) $$\begin{array}{cc} \; & R\leq R_{E,g^{\prime }},\;\mathrm{if}\;\;{\tilde{l}}_{g}={\hat{l}}_{g^{\prime }},\;\;g\in G_{U},\;g^{\prime }\in G_{E}, \end{array}$$

(31e) $$\begin{array}{cc} \; & \mathrm{tr}\left(E_{i}^{H}W_{g}W_{g}^{H}E_{i}\right)=0,\;\;\mathrm{if}\;\;c_{i,{\tilde{l}}_{g}}=u_{i,{\tilde{l}}_{g}}=0,\;i\in N_{E},\;g\in G_{U}, \end{array}$$

(31f) $$\begin{array}{cc} \; & p_{C}\left(A\right)\leq P_{C}, \end{array}$$

(31g) $$\begin{array}{cc} \; & p_{E,i}\left(W\right)\leq P_{E,i},\;\;i\in N_{E}, \end{array}$$

with $R_{E}≜{\left\{R_{E,g}\right\}}_{g\in G_{E}}$. In (Section 4.4), the function $p_{\mathrm{total}}\left(A,W\right)$ is equal to the total power consumption of the network and is defined as

(32) $$p_{\mathrm{total}}\left(A,W\right)=p_{C}\left(A\right)+\eta_{C}+\sum_{i\in N_{E}}\left(p_{E,i}\left(W\right)+\eta_{E,i}\right),$$

with $p_{C}\left(A\right)=\sum_{g\in G_{E}}\mathrm{tr}\left(A_{g}A_{g}^{H}\right)$ and $p_{E,i}\left(W\right)=\sum_{g\in G_{U}}\mathrm{tr}\left(E_{i}^{H}W_{g}W_{g}^{H}E_{i}\right)$ being the RF transmision powers at the CP and EN *i*, respectively. The constraint (31b), which is equivalent to (29), imposes that the requested files are reliably decoded at the UEs and the constraint (31c) guarantees that all the multicast messages on the wireless fronthaul link are successfully decoded by the requesting ENs. As the rate of each requested file cannot exceed the rate at which the file is communicated over the fronthaul link, the constraint (31d) is imposed. The condition (31e) indicates that each EN can precode only the files that are stored in its cache or received from the CP over the fronthaul link. The constraints (31f) and (31g) stand for the CP and per-EN transmision power constraints.

The formulated problem (31) is non-convex. However, since it has a similar form to that of the problem (14) defined for the soft-transfer scheme, we can tackle (31) in a similar manner to that discussed in Section 3.5: We replace the objective fractional function in (31a) with a new variable Θ, which is constrained by

(33) $$\ln\Theta \leq \ln R-\ln\;p_{\mathrm{total}}\left(A,W\right).$$

This yields the following equivalent problem:

(34a) $$\begin{array}{cc} \underset{A,W,R,R_{E},\Theta }{\mathrm{maximize}}\;\; & \Theta \end{array}$$

(34b) $$\begin{array}{cc} s.t.\;\; & \ln\Theta \leq \ln R-\ln\;p_{\mathrm{total}}\left(A,W\right),\\ \; & (31b)–(31g). \end{array}$$

As in Section 3.5, from (34), we can obtain a DC problem by defining a change of variables ${\tilde{A}}_{g}=A_{g}A_{g}^{H}$ and ${\tilde{W}}_{g}=W_{g}W_{g}^{H}$ and relaxing the non-convex constraints $\mathrm{rank}\left({\tilde{A}}_{g}\right)\leq d_{E,g}$ and $\mathrm{rank}\left({\tilde{W}}_{g}\right)\leq d_{U,g}$. Thus, we propose to tackle the DC problem by deriving a CCCP-based iterative algorithm, which is detailed in Algorithm 2, followed by the projections $A_{g}\leftarrow Q_{d_{E,g}}\left({\tilde{A}}_{g}\right)\;S_{d_{E,g}}{\left({\tilde{A}}_{g}\right)}^{1/2}$ and $W_{g}\leftarrow Q_{d_{U,g}}\left({\tilde{W}}_{g}\right)\;S_{d_{U,g}}{\left({\tilde{W}}_{g}\right)}^{1/2}$. Similar to Algorithm 1, the complexity of Algorithm 2 can be expressed as $O(N_{\mathrm{itr}}\sqrt{N_{\mathrm{const}}}\log\left(N_{\mathrm{const}}/\epsilon \right))$, where the number $N_{\mathrm{const}}$ of constraints for the convex problem (A2) is randomly given, depending on the request profiles of the UEs and the cached contents of the ENs. It is guaranteed that $N_{\mathrm{const}}$ is bounded as $N_{\mathrm{const}}\leq N_{U}^{2}+N_{E}N_{U}+2N_{E}+N_{U}+2$, where the upper bound is approximated, for sufficiently large $N_{U}$ and $N_{E}$, as $N_{U}\left(N_{U}+N_{E}\right)$.
**Algorithm 2** CCCP-based algorithm for problem (34).**1.** Initialize the variables $\Theta^{\prime },{\tilde{A}}^{\prime },$ and ${\tilde{W}}^{\prime }$ that satisfy the constraints of the problem (34). **2.** Update $\Theta^{\prime \prime },{\tilde{A}}^{\prime \prime }$ and ${\tilde{W}}^{\prime \prime }$ as an optimal solution of the (convex) problem (A2) in Appendix A. **3.** Stop if a convergence criterion is satisfied. Otherwise, set $\Theta^{\prime }\leftarrow \Theta^{\prime \prime },{\tilde{A}}^{\prime }\leftarrow {\tilde{A}}^{\prime \prime }$ and ${\tilde{W}}^{\prime }\leftarrow {\tilde{W}}^{\prime \prime }$ and go back to Step 2.

## 5. Numerical Results

In this section, we validate the effectiveness of the proposed energy-efficient designs under the soft-transfer and hard-transfer fronthauling strategies proposed in Section 3 and Section 4, respectively, through numerical results. Throughout the section, we assume that the positions of the ENs and UEs are uniformly distributed within a circular region of radius 100 m and the CP is located at the center. Furthermore, we assume Rayleigh fading for all channel elements of the fronthaul ${\left\{H_{i}\right\}}_{i\in N_{E}}$ and edge links ${\left\{G_{k,i}\right\}}_{k\in N_{U},i\in N_{E}}$, and adopt the path-loss model $1/(1+{\left(\mathrm{distance}/D_{0}\right)}^{\beta })$ considered in [13,15,39], where we set $D_{0}=30$ m and β=3. We define the SNRs of the fronthaul and edge links as $P_{C}/\sigma_{E}^{2}$ and $P_{E}/\sigma_{U}^{2}$, respectively; where we assume that every EN uses the same transmission power $P_{E}$, i.e., $P_{E,i}=P_{E}$ for all $i\in N_{E}$. Also, we set the circuit powers $\eta_{C}$ and $\eta_{E,i}$ of the CP and each EN to be 10 dB larger than the noise powers, $\sigma_{E}^{2}$ and $\sigma_{U}^{2}$, of the corresponding links.

The number of files in the library F is set to L=10, and the Zipf’s distribution is considered for the popularity of the files. That is, the probability $\mathrm{Pr}[l_{k}=l]$ that UE *k* requests the file $f_{l}$ is given as $\mathrm{Pr}\left[l_{k}=l\right]=cl^{-\gamma }$ with $c=1/(\sum_{l=1}^{L}l^{-\gamma })$. We set the constant γ (which controls the skewness of the popularity among the files) as γ=1. Assuming that $B_{i}=B$ for all $i\in N_{E}$, we define the fractional cache size as $\mu =B/S_{\mathrm{library}}\in \left[0,1\right]$: μ=0 means that the ENs do not have caching functionality, and μ=1 indicates that the full library F is available at all the ENs. For a partial caching case, with 0<μ<1, we consider a random caching strategy, in which each EN pre-fetches $\left\lfloor \mu L\right\rfloor$ files, randomly chosen from the *L* files in the library F.

**Figure 3:**
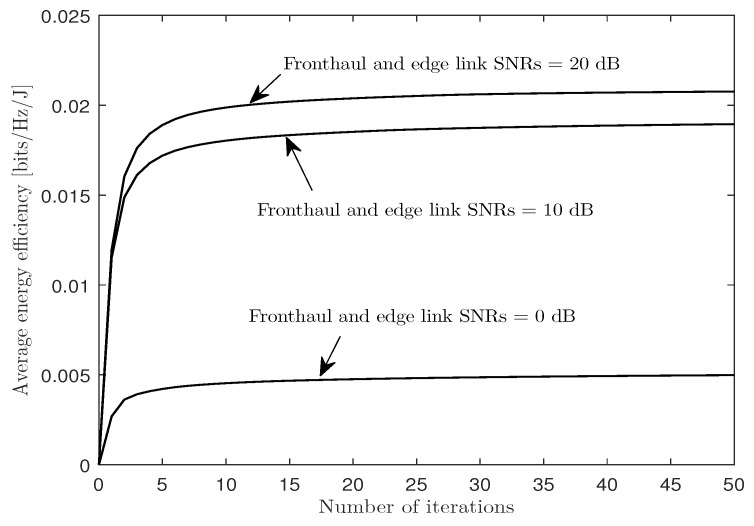
Average EE Θ versus the number of iterations with the soft-transfer fronthauling strategy for a cache-aided C-RAN system with $n_{C}=8$, $N_{E}=N_{U}=4$, $n_{E,i}=n_{U,k}=2$, μ=0.3, and $P_{C}/\sigma_{E}^{2}=P_{E}/\sigma_{U}^{2}\in \left\{0,10,20\right\}$ dB.

We first observe the convergence behavior of the proposed CCCP-based iterative algorithm for the soft-transfer fronthauling scheme in Section 3 (i.e., Algorithm 1) by plotting, in Figure 3, the average EE performance Θ with respect to the number of iterations for a cache-aided C-RAN system with $n_{C}=8$, $N_{E}=N_{U}=4$, $n_{E,i}=n_{U,k}=2$, μ=0.3, and $P_{C}/\sigma_{E}^{2}=P_{E}/\sigma_{U}^{2}\in \left\{0,10,20\right\}$ dB. The figure shows that, regardless of SNR values, the algorithm converged within a few tens of iterations.

**Figure 4:**
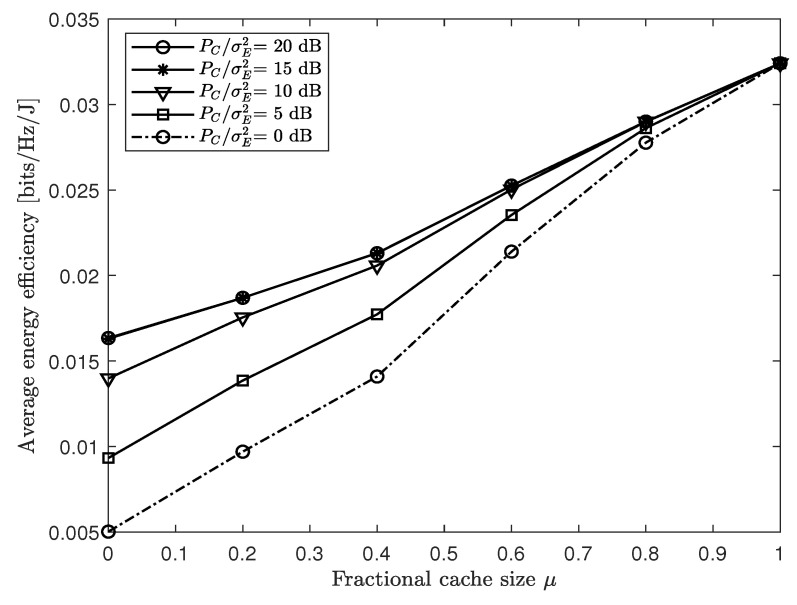
Average EE Θ versus the fractional cache size μ with the soft-transfer fronthauling strategy (Section 3) for a cache-aided C-RAN system with $n_{C}=8$, $N_{E}=N_{U}=4$, $n_{E,i}=n_{U,k}=2$, $P_{C}/\sigma_{E}^{2}\in \left\{0,5,10,15,20\right\}$ dB, and $P_{E}/\sigma_{U}^{2}=20$ dB.

In Figure 4, we plot the average EE performance Θ of the proposed energy-efficient design with the soft-transfer fronthauling strategy proposed in Section 3 versus the fractional cache size μ for a cache-aided C-RAN system with $n_{C}=8$, $N_{E}=N_{U}=4$, $n_{E,i}=n_{U,k}=2$, $P_{C}/\sigma_{E}^{2}\in \left\{0,5,10,15,20\right\}$ dB, and $P_{E}/\sigma_{U}^{2}=20$ dB. The figure shows that, as the ENs could pre-fetch more popular contents to the local caches, the overhead on the wireless fronthaul link was reduced, which led to better EE performance of the overall network. Furthermore, it is noted that the impact of the cache size was more pronounced when the fronthaul SNR $P_{C}/\sigma_{E}^{2}$ was smaller, owing to the fact that, with lower fronthaul SNR level, the fronthaul overhead became a performance bottleneck. Hence, equipping ENs with caches will be more helpful. In a similar vein, for the full caching case (μ=1), varying the fronthaul SNR did not affect the performance.

**Figure 5:**
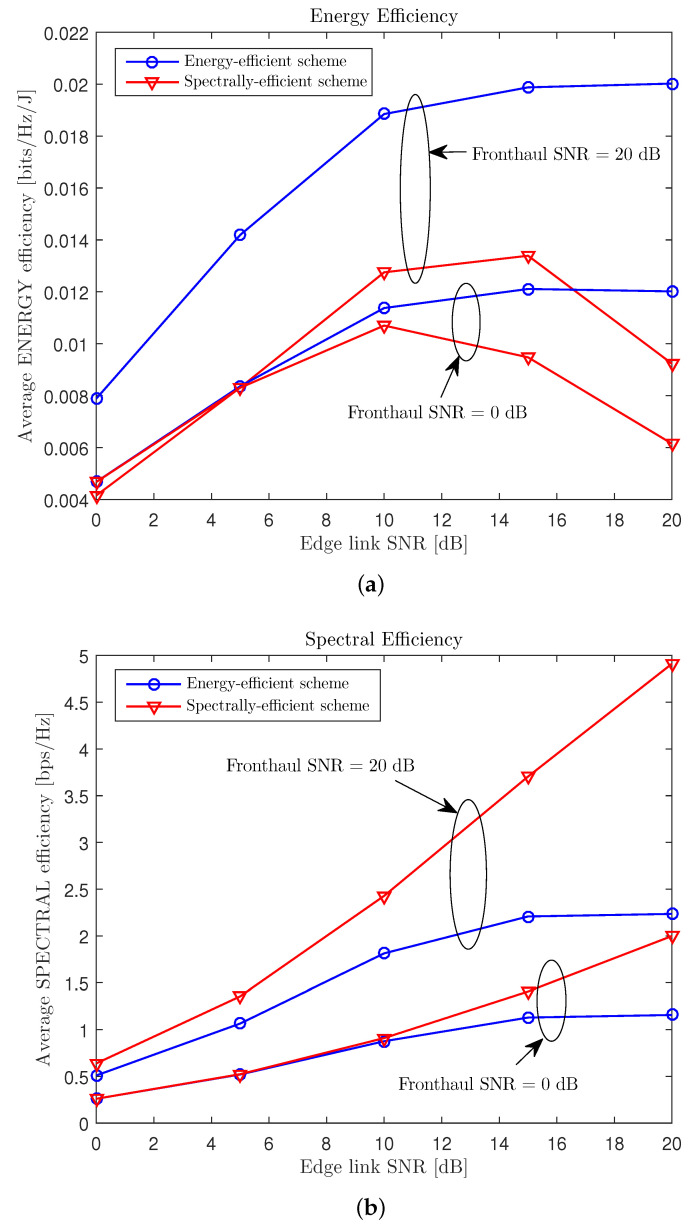
Average EE Θ and SE *R* versus the edge link SNR $P_{E}/\sigma_{U}^{2}$ with the “soft-transfer” fronthauling strategy (Section 3) for a cache-aided C-RAN system with $n_{C}=8$, $N_{E}=N_{U}=4$, $n_{E,i}=n_{U,k}=2$, μ=0.3, and $P_{C}/\sigma_{E}^{2}\in \left\{0,20\right\}$ dB ((**a**) EE; (**b**) SE).

In Figure 5, we show the average EE Θ, as well as the spectral efficiency (SE) *R*, of the proposed energy-efficient soft-transfer scheme in Section 3 with respect to the edge link SNR $P_{E}/\sigma_{U}^{2}$ for a cache-aided C-RAN system with $n_{C}=8$, $N_{E}=N_{U}=4$, $n_{E,i}=n_{U,k}=2$, μ=0.3, and $P_{C}/\sigma_{E}^{2}\in \left\{0,20\right\}$ dB. To validate the importance of the energy-efficient design, we also plot the EE and SE performance of the spectrally-efficient design, which solves the problem (14) replacing the EE objective function with the spectral efficiency *R*. From Figure 5, we observe that, when the fronthaul and edge links had small SNRs, the energy- and spectrally-efficient schemes provided similar EE and SE performances, which means that the energy-efficient solution tended to use full transmission power at the CP and the ENs to maximize the SE metric. In contrast, when the fronthaul or edge link had a sufficiently large SNR, it would be better to use only partial transmission power at the CP or the ENs to achieve a better EE performance, at the cost of the SE value.

**Figure 6:**
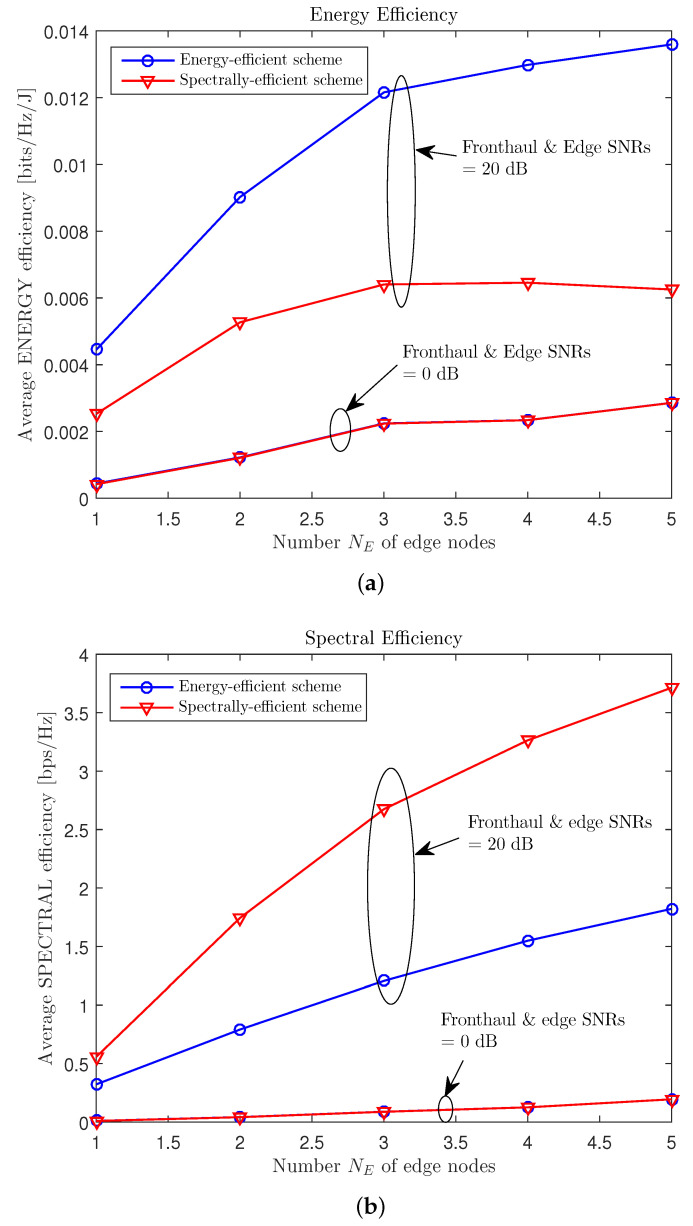
Average EE Θ and SE *R* versus the number $N_{E}$ of edge nodes with the “soft-transfer” fronthauling strategy (Section 3) for a cache-aided C-RAN system with $n_{C}=4$, $N_{U}=4$, $n_{E,i}=2$, $n_{U,k}=1$, μ=0.3, and $P_{C}/\sigma_{E}^{2}=P_{E}/\sigma_{U}^{2}\in \left\{0,20\right\}$ dB ((**a**) EE; (**b**) SE).

Figure 6 compares the average EE Θ and SE *R* of the energy- and spectrally-efficient schemes for various numbers $N_{E}$ of ENs, with $n_{C}=4$, $N_{U}=4$, $n_{E,i}=2$, $n_{U,k}=1$, μ=0.3, and $P_{C}/\sigma_{E}^{2}=P_{E}/\sigma_{U}^{2}\in \left\{0,20\right\}$ dB. We can see that, regardless of the number of edge nodes, the energy-efficient and spectrally-efficient schemes showed almost same EE and SE performance when the SNRs of the fronthaul and edge links were low. However, when the SNRs were sufficiently large, the energy-efficient scheme showed a notable gain, which increased with the number of ENs, over the spectrally-efficient scheme. This suggests that, when there were many ENs and the SNRs were large, the spectrally-efficient scheme encouraged all of the ENs to use full transmission power, while the energy-efficient scheme allocated only the necessary level of transmission power to the ENs.

**Figure 7:**
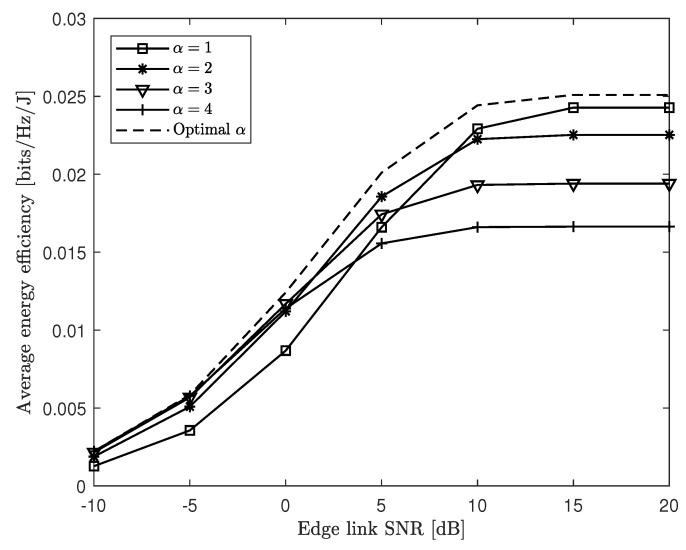
Average EE Θ versus the edge link SNR $P_{E}/\sigma_{U}^{2}$ with the proposed “hard-transfer” fronthauling strategy (Section 4) for a cache-aided C-RAN system with $n_{C}=8$, $N_{E}=N_{U}=4$, $n_{E,i}=n_{U,k}=2$, μ=0.3, and $P_{C}/\sigma_{E}^{2}\in 20$ dB.

Figure 7 plots the average EE performance Θ of the proposed energy-efficient hard-transfer scheme in Section 4 versus the edge link SNR $P_{E}/\sigma_{U}^{2}$ for a cache-aided C-RAN system with $n_{C}=8$, $N_{E}=N_{U}=4$, $n_{E,i}=n_{U,k}=2$, μ=0.3, and $P_{C}/\sigma_{E}^{2}=20$ dB. In the figure, we include the performance for the cases of all possible connectivity levels $\alpha \in \{1,\ldots ,N_{E}\}$, as well as for the case when the optimal α is chosen, corresponding to the largest EE for each channel realization. As expected, in the regime of low edge SNRs, the overall performance was limited by the edge link, rather than the fronthaul link. Therefore, it is desirable to use large connectivity level α to maximize the co-operation gain of the ENs. On the other hand, when the edge SNR is large enough, the limitation at the fronthaul link becomes dominant; hence, it would be better to decrease the connectivity level α to reduce the fronthaul overhead. We also note that using the best connectivity level α in adaptation to the instantaneous CSI yields further improvement, particularly in the intermediate edge SNR levels.

**Figure 8:**
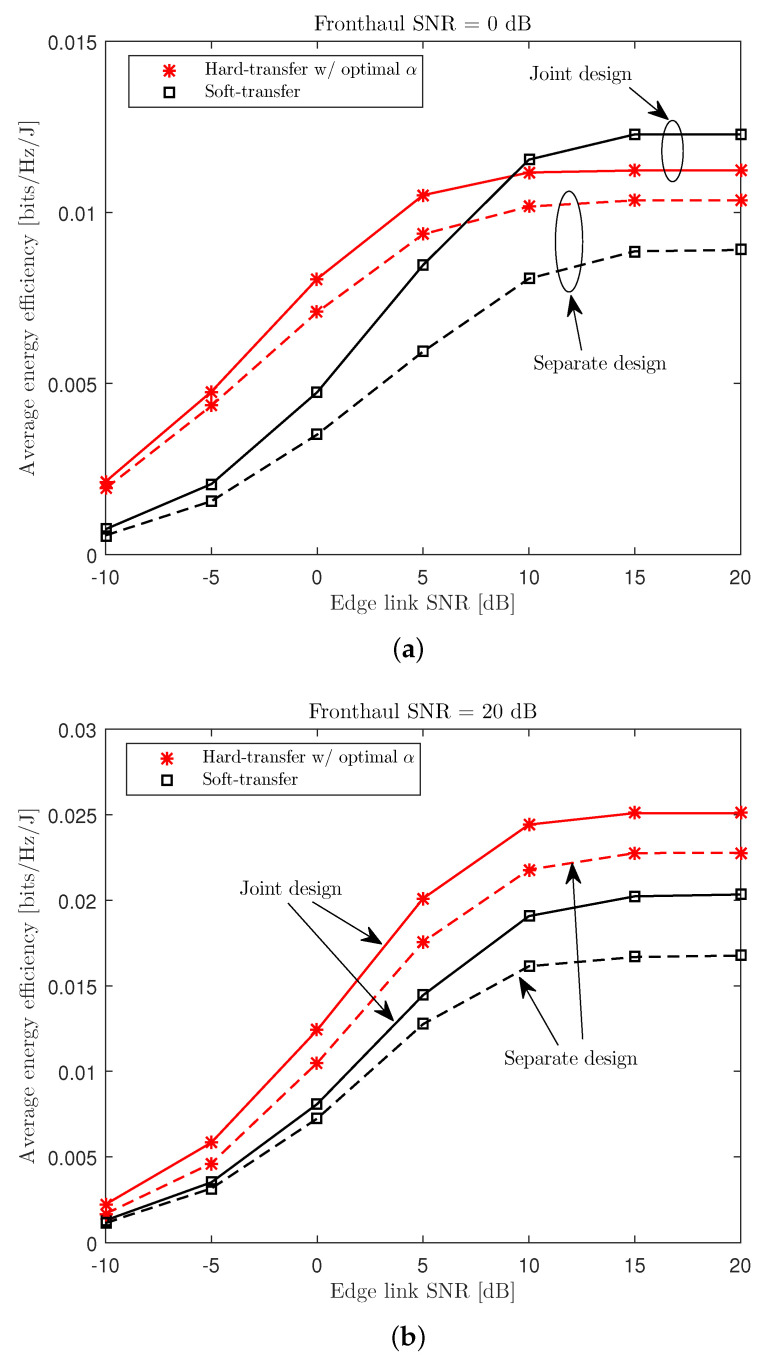
Average EE Θ versus the edge link SNR $P_{E}/\sigma_{U}^{2}$ with the soft- and hard-transfer fronthauling strategies for a cache-aided C-RAN system with $n_{C}=8$, $N_{E}=N_{U}=4$, $n_{E,i}=n_{U,k}=2$, μ=0.3, and $P_{C}/\sigma_{E}^{2}\in \left\{0,20\right\}$ dB ((**a**) $P_{C}/\sigma_{E}^{2}=0$ dB; (**b**) $P_{C}/\sigma_{E}^{2}=20$ dB).

In Figure 8, we compare the average EE performance Θ of the soft-transfer and hard-transfer fronthauling schemes for a cache-aided C-RAN system with $n_{C}=8$, $N_{E}=N_{U}=4$, $n_{E,i}=n_{U,k}=2$, μ=0.3, and $P_{C}/\sigma_{E}^{2}=20$ dB. For the hard-transfer scheme, we used the optimal connectivity level α which gave the best performance for each channel sample. We can observe, from the graph, that the hard-transfer fronthauling scheme achieved a better EE performance than the soft-transfer scheme in overall system environments. This supports the fact that the hard-transfer scheme is more effective in utilizing the multicasting opportunity in the wireless fronthaul link, while the soft-transfer scheme starts with orthogonalizing the wireless fronthaul links across the ENs by means of the SDMA fronthaul precoding. However, it should be noted that, when the fronthaul link SNR was significantly smaller than that of the edge link, the soft-transfer scheme (whereby the fronthaul link carried compressed information) showed a better EE performance than the hard-transfer scheme. In Figure 8, we also compare the performance of the proposed joint design of fronthaul and edge links with those of simpler separate design methods. The separate schemes, first, design the fronthaul-related variables with the goal of maximizing the EE of the fronthaul transmission and, then, optimize the remaining variables related to the edge link transmission. The figure shows that the performance gain of the joint design was significant for both the soft-transfer and hard-transfer fronthauling strategies and grew with the edge link SNR.

**Figure 9:**
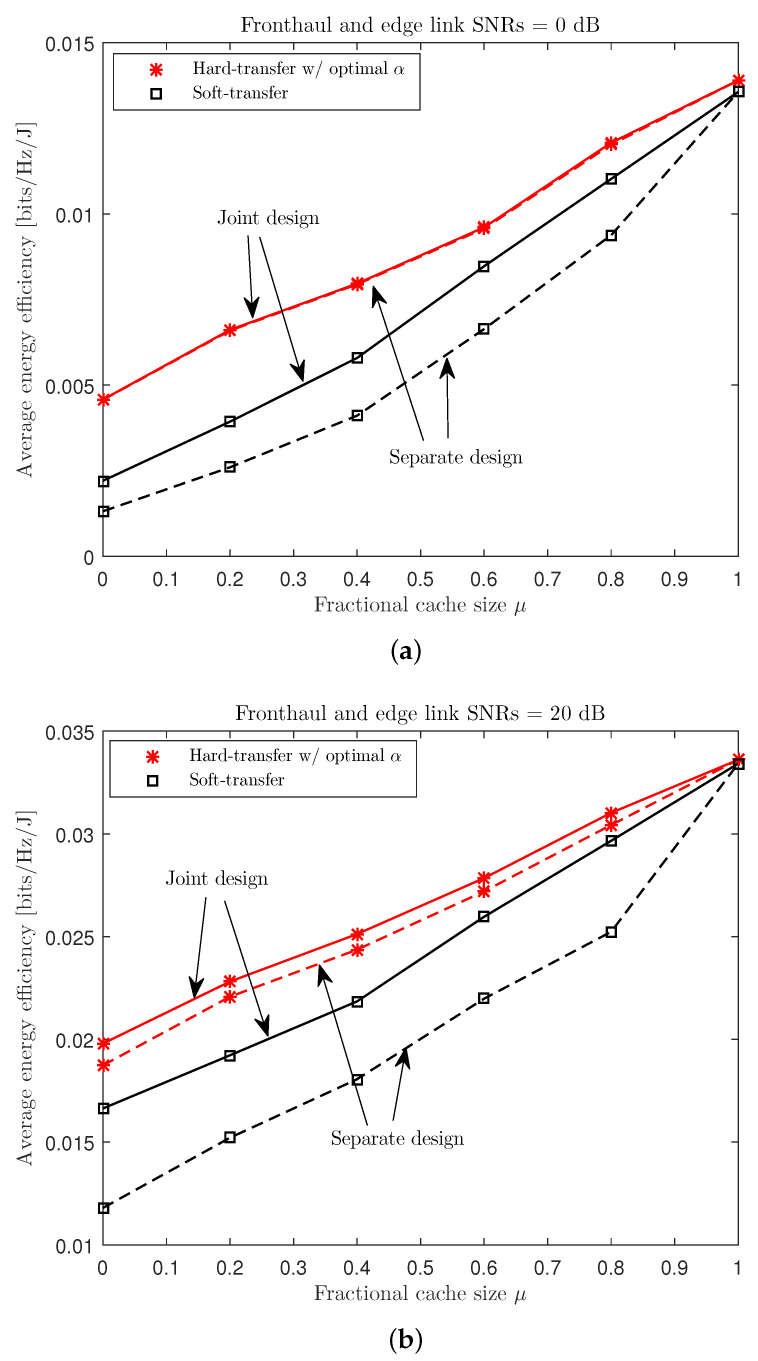
Average EE Θ versus the fractional cache size μ with the soft- and hard-transfer fronthauling strategies for a cache-aided C-RAN system with $n_{C}=8$, $N_{E}=N_{U}=4$, $n_{E,i}=n_{U,k}=2$, and $P_{C}/\sigma_{E}^{2}=P_{E}/\sigma_{U}^{2}\in \left\{0,20\right\}$ dB ((**a**) $P_{C}/\sigma_{E}^{2}=P_{E}/\sigma_{U}^{2}=0$ dB; (**b**) $P_{C}/\sigma_{E}^{2}=P_{E}/\sigma_{U}^{2}=20$ dB).

Lastly, in Figure 9, we compare the average EE performance Θ of the schemes considered in Figure 8, with respect to the fractional cache size μ for a cache-aided C-RAN with $n_{C}=8$, $N_{E}=N_{U}=4$, $n_{E,i}=n_{U,k}=2$, and $P_{C}/\sigma_{E}^{2}=P_{E}/\sigma_{U}^{2}\in \left\{0,20\right\}$ dB. As the soft- and hard-transfer schemes differ only in terms of the fronthaul usage, the EE values of both schemes approached the same value as μ increased (i.e., ENs pre-fetched more content and, hence, the amount of traffic over the fronthaul was reduced). In a similar vein, the advantage of the proposed joint design became minor for sufficiently large μ.

## 6. Conclusions

We have discussed the energy-efficient joint design of the fronthaul and edge transmission strategies for a cache-aided C-RAN system with a wireless fronthaul link. Specifically, we have tackled the problem of maximizing the EE metric under both the soft- and hard-transfer fronthauling strategies. We have converted the formulated non-convex optimization problems into DC problems by means of change of variables and rank relaxation and tackled the resulting problems using the CCCP approach. Through numerical results, we have validated the impact of caching functionality and the advantages of an energy-efficient design over a spectrally-efficient scheme, particularly at high fronthaul and edge SNRs. It was also observed that, in the overall simulated cases, the hard-transfer scheme can better utilize the multicasting opportunity of the wireless fronthaul transmission, as compared to the soft-transfer scheme. Furthermore, we have verified that the importance of the joint design of the fronthaul and edge links is more significant when the fronthaul and edge links have a larger SNR, or when the ENs pre-fetch less content.

## Figures and Tables

**Table 1:** Summary of the main differences between this and related studies on C-RAN systems.

Features		References
Edge caching	No edge caching	[4,5,6,7,8,9,10,11,12,13,14,15,16,17,18,31,34,39]
	With edge caching	[21,22,23,24,25,26,27,28,40], this work
Fronthaul links	Wired fronthaul	[4,5,6,7,8,9,10,21,23,24,25,26,27,28,31,34,39,40]
	Wireless fronthaul	[11,12,13,14,15,16,17,18], this work
Design goal	Max. spectral efficiency	[4,5,6,7,8,9,10,13,14,15,16,17,18,26,39]
	Min. delivery latency	[23,27,40]
	Min. power or network cost	[11,12,24,25,28]
	Max. energy efficiency	[21,31,34], this work
Fronthauling strategy	Soft-transfer	[4,5,7,9,10,12,13,15,39]
	Hard-transfer	[11,14,16,17,18,21,24,25,40]
	Both	[6,8,23,26,27,28,31,34], this work

## Appendix A

The convex problem that is solved at Step 2 of Algorithm 1 is defined as

(A1) $$\begin{array}{cc} \underset{\tilde{F},\tilde{T},\Omega ,R,\Theta }{\mathrm{maximize}}\;\; & \;\;\Theta \end{array}$$

$$\begin{array}{cc} s.t.\;\; & \tilde{\psi }\left(\Theta ,\tilde{F},\tilde{T},\Omega ,\Theta^{\prime },{\tilde{F}}^{\prime },{\tilde{T}}^{\prime },\Omega^{\prime }\right)\leq \ln R,\\ \; & R\;\leq \;{\tilde{f}}_{U,g,k}\left(\tilde{T},\Omega ,{\tilde{T}}^{\prime },\Omega^{\prime }\right),\;g\in G_{U},\;k\in N_{U,g},\\ \; & {\tilde{g}}_{i}\left(\tilde{T},\Omega ,{\tilde{T}}^{\prime },\Omega^{\prime }\right)\leq {\tilde{f}}_{E,i}\left(\tilde{F},{\tilde{F}}^{\prime }\right),\;\;i\in N_{E},\\ \; & p_{C}\left(\tilde{F}\right)\leq P_{C},\\ \; & p_{E,i}\left(\tilde{T},\Omega \right)\leq P_{E,i},\;\;i\in N_{E}, \end{array}$$

where we define the functions

$$\begin{array}{cc} \; & \tilde{\psi }\left(\Theta ,\tilde{F},\tilde{T},\Omega ,\Theta^{\prime },{\tilde{F}}^{\prime },{\tilde{T}}^{\prime },\Omega^{\prime }\right)\\ \; & ≜\varphi \left(\Theta ,\Theta^{\prime }\right)+\varphi \left(p_{\mathrm{total}}\left(\tilde{F},\tilde{T},\Omega \right),p_{\mathrm{total}}\left({\tilde{F}}^{\prime },{\tilde{T}}^{\prime },\Omega^{\prime }\right)\right),\\ \; & {\tilde{f}}_{U,g,k}\left(\tilde{T},\Omega ,{\tilde{T}}^{\prime },\Omega^{\prime }\right)\\ \; & ≜\log_{2}\det\left(\sum_{g^{\prime }\in G_{U}}G_{k}{\tilde{T}}_{g^{\prime }}G_{k}^{H}+G_{k}\bar{\Omega }G_{k}^{H}+\sigma_{U}^{2}I_{n_{U,k}}\right)\\ \; & -\varphi \left(\begin{array}{c} \sum_{g^{\prime }\in G_{U}\;\setminus \left\{g\right\}}G_{k}{\tilde{T}}_{g^{\prime }}G_{k}^{H}+G_{k}\bar{\Omega }G_{k}^{H}+\sigma_{U}^{2}I_{n_{U,k}},\\ \sum_{g^{\prime }\in G_{U}\;\setminus \left\{g\right\}}G_{k}{\tilde{T}}_{g^{\prime }}^{\prime }G_{k}^{H}+G_{k}{\bar{\Omega }}^{\prime }G_{k}^{H}+\sigma_{U}^{2}I_{n_{U,k}} \end{array}\right),\\ \; & {\tilde{f}}_{E,i}\left(\tilde{F},{\tilde{F}}^{\prime }\right)≜\log_{2}\det\left(\sum_{j\in N_{E}}H_{i}{\tilde{F}}_{j}H_{i}^{H}+\sigma_{E}^{2}I_{n_{E,i}}\right)\\ \; & -\varphi \left(\begin{array}{c} \sum_{j\in N_{E}\setminus \left\{i\right\}}H_{i}{\tilde{F}}_{j}H_{i}^{H}+\sigma_{E}^{2}I_{n_{E,i}},\\ \sum_{j\in N_{E}\setminus \left\{i\right\}}H_{i}{\tilde{F}}_{j}^{\prime }H_{i}^{H}+\sigma_{E}^{2}I_{n_{E,i}} \end{array}\right),\\ \; & {\tilde{g}}_{i}\left(\tilde{T},\Omega ,{\tilde{T}}^{\prime },\Omega^{\prime }\right)\\ \; & ≜\varphi \left(\begin{array}{c} \sum_{g\in G_{U}}{\bar{c}}_{i,{\tilde{l}}_{g}}E_{i}^{H}{\tilde{T}}_{g}E_{i}+\Omega_{i},\\ \sum_{g\in G_{U}}{\bar{c}}_{i,{\tilde{l}}_{g}}E_{i}^{H}{\tilde{T}}_{g}^{\prime }E_{i}+\Omega_{i}^{\prime } \end{array}\right)-\log_{2}\det\left(\Omega_{i}\right), \end{array}$$

with the notation $\varphi \left(A,B\right)≜\log_{2}\det\left(B\right)+\mathrm{tr}\left(B^{-1}\left(A-B\right)\right)$.

Also, at Step 2 of Algorithm 2, we solve the following convex problem:

(A2) $$\begin{array}{cc} \underset{\tilde{A},\tilde{W},R,R_{E}}{\mathrm{maximize}}\;\; & \Theta \end{array}$$

$$\begin{array}{cc} s.t.\;\; & \tilde{\psi }\left(\Theta ,\tilde{A},\tilde{W},\Theta^{\prime },{\tilde{A}}^{\prime },{\tilde{W}}^{\prime }\right)\leq \ln R,\\ \; & R\leq {\tilde{f}}_{U,g,k}\left(\tilde{W},{\tilde{W}}^{\prime }\right),\;g\in G_{U},\;k\in N_{U,g},\\ \; & R_{E,g}\leq {\tilde{f}}_{E,g,i}\left(\tilde{A},{\tilde{A}}^{\prime }\right),\;\;g\in G_{E},\;i\in N_{E,g},\\ \; & R\leq R_{E,g^{\prime }},\;\mathrm{if}\;\;{\tilde{l}}_{g}={\hat{l}}_{g^{\prime }},\;\;g\in G_{U},\;g^{\prime }\in G_{E},\\ \; & \mathrm{tr}\left(E_{i}^{H}{\tilde{W}}_{g}E_{i}\right)=0,\;\mathrm{if}\;\;c_{i,{\tilde{l}}_{g}}=u_{i,{\tilde{l}}_{g}}=0,\;\;i\in N_{E},\;g\in G_{U},\\ \; & p_{C}\left(\tilde{A}\right)\leq P_{C},\\ \; & p_{E,i}\left(\tilde{W}\right)\leq P_{E,i},\;\;i\in N_{E}. \end{array}$$

where we define the functions

$$\begin{array}{cc} \; & \tilde{\psi }\left(\Theta ,\tilde{A},\tilde{W},\Theta^{\prime },{\tilde{A}}^{\prime },{\tilde{W}}^{\prime }\right)\\ \; & ≜\varphi \left(\Theta ,\Theta^{\prime }\right)+\varphi \left(p_{\mathrm{total}}\left(\tilde{A},\tilde{W}\right),p_{\mathrm{total}}\left({\tilde{A}}^{\prime },{\tilde{W}}^{\prime }\right)\right),\\ \; & {\tilde{f}}_{U,g,k}\left(\tilde{W},{\tilde{W}}^{\prime }\right)\\ \; & ≜\log_{2}\det\left(\sum_{g^{\prime }\in G_{U}}G_{k}{\tilde{W}}_{g^{\prime }}G_{k}^{H}+\sigma_{U}^{2}I_{n_{U,k}}\right)\\ \; & -\varphi \left(\begin{array}{c} \sum_{g^{\prime }\in G_{U}\setminus \left\{g\right\}}G_{k}{\tilde{W}}_{g^{\prime }}G_{k}^{H}+\sigma_{U}^{2}I_{n_{U,k}},\\ \sum_{g^{\prime }\in G_{U}\setminus \left\{g\right\}}G_{k}{\tilde{W}}_{g^{\prime }}^{\prime }G_{k}^{H}+\sigma_{U}^{2}I_{n_{U,k}} \end{array}\right),\\ \; & {\tilde{f}}_{E,g,i}\left(\tilde{A},{\tilde{A}}^{\prime }\right)\\ \; & ≜\log_{2}\det\left(\sum_{m\in G_{E}}{\bar{c}}_{i,{\hat{l}}_{m}}H_{i}{\tilde{A}}_{m}H_{i}^{H}+\sigma_{E}^{2}I_{n_{E,i}}\right)\\ \; & -\varphi \left(\begin{array}{c} \sum_{m\in G_{E}\setminus \left\{g\right\}}{\bar{c}}_{i,{\hat{l}}_{m}}H_{i}{\tilde{A}}_{m}H_{i}^{H}+\sigma_{E}^{2}I_{n_{E,i}},\\ \sum_{m\in G_{E}\setminus \left\{g\right\}}{\bar{c}}_{i,{\hat{l}}_{m}}H_{i}{\tilde{A}}_{m}^{\prime }H_{i}^{H}+\sigma_{E}^{2}I_{n_{E,i}} \end{array}\right). \end{array}$$
